# The criminalisation of miscarriage associated with illicit substance consumption whilst pregnant

**DOI:** 10.1177/00258024221140666

**Published:** 2022-12-14

**Authors:** Josephine Taylor, Sajeel A Shah, Nikolas P Lemos

**Affiliations:** Cameron Forensic Medical Sciences, Centre for Clinical Pharmacology and Precision Medicine, William Harvey Research Institute, Barts and The London School of Medicine and Dentistry, Queen Mary University of London, London, UK

The official rights of a foetus continue to be an unending legal and philosophical conversation. Yet newer discussion has emerged on the convictions of mothers for their spontaneous loss of pregnancy.

In October 2021, the state of Oklahoma convicted Brittney Poolaw of first-degree manslaughter after the miscarriage of her 15 to 17-week-old foetus and sentenced her to four years in prison. After admitting to methamphetamine and marijuana use during pregnancy, trace quantities of these substances were found in the foetus’ brain and liver on post-mortem.^1^ However, the report also cited evidence of ‘congenital abnormality … placental abruption and chorioamnionitis’, all deemed by the medical examiner as ‘conditions contributing’ to miscarriage.^2,3^

As 12–24% of recognised pregnancies result in miscarriage,^4^ there is no consistent, investigatory process to consider these as homicides. Currently, a growing adversarial relationship between pregnant women and their foetuses is challenging women's rights. Between 2006 and 2020, the National Advocates for Pregnant Women (NAPW) identified 1254 cases where women *but for* their pregnancy, would not have been subject to legal charges.^5^ Expanding awareness of prenatal harms questions the expectation for pregnant women to conform to new, scientific discoveries. Can a woman be wrong ‘for drinking coffee or exercising too little, each of which could pose some risk to a fetus’?.^6^

There is legal contention on the shifting grounds convicting Poolaw. By ruling miscarriage as manslaughter, the intentional abortion of a foetus could constitute a murder. However, at the time, Oklahoma permitted abortion up to 22 weeks of gestation. Poolaw's 17-week-old foetus was not viable, and a termination of pregnancy would be legal. Furthermore, criminalising substance abuse whilst pregnant may conversely *not* protect the health of mother and child as fear of punishment creates a healthcare barrier.^7^ In Oklahoma and 24 other states, healthcare providers must report suspected prenatal drug use to police.^3^ Yet pregnancy could catalyse a change in mindset, producing a window of opportunity for cessation of recreational drug use that should be harnessed. Some argue incarceration for addiction is the wrong approach for a classified disease, considering the successful approach of Portugal to prioritise the ‘psychosocial vulnerability of high-risk users’ by decriminalising drug use in 2000.^8^

*Roe v Wade 1973* used to shape a legal framework in the US, meaning states could not outlaw abortion before the point of foetal viability. States could restrict abortion before that point but not inflict ‘undue burden’ on women seeking abortions (*Planned Parenthood v Casey 1992*). In May 2022, Oklahoma signed the Heartbeat Act – Senate Bill 1503, one of the most restrictive bans across the US. It sidestepped *Roe v Wade* by using civilian enforcement rather than state officials to criminalise abortion if cardiac activity can be detected.^9^ Now the legal basis of *Roe v Wade* has been overturned by the current Mississippi case in the Supreme Court and loopholes are no longer needed to impede abortion access. The Unborn Victims of Violence Act 2004 recognises foetuses as a legal victim so women can be prosecuted for feticide, chemical endangerment and murder.^5^ Cases such as Poolaw's – guilty of misdemeanour manslaughter by drug possession and consumption, could become a recurring scenario.

Conflicting scientific opinion compromises understanding of the effects of foetal exposure to drug use. NAPW strongly states that ‘none of the criminalised drugs are teratogens’ or have not shown to be ‘effective at causing pregnancy loss’.^10^ Studies reveal negative effects of cocaine, alcohol and opiates on birthweight, neurodevelopment and the understanding of neonatal abstinence syndrome.^11–14^ Yet cannabis and methamphetamine are two drugs particularly lacking research.^15^ Furthermore, correlation with adverse outcomes does not infer causation. One study found ‘the effects of poverty, poor diet, and tobacco use … to be as harmful or more harmful than the drug use itself’.^16^ So, whilst exposing the necessitation for further appraisal, current science does not appear to categorically link Poolaw's miscarriage to her substance use.

There may be culpability in knowingly poisoning a foetus but the definition and evidence of ‘knowingly’ and ‘poisoning’ is elusive. For Poolaw, this ambiguity encourages NAPW to challenge the justification of her charges. The constitutional right to legal abortion in the USA was never wholly accessible due to federal policies and state restrictions, yet now that overarching right is gone, law-making authority is delegated to individual states. Only some of these are predicted to protect access to abortions and women's bodily autonomy. Alongside the discourse defining a foetus’ legal independence and differing attitudes towards substance use, the resulting dynamic legal and socioeconomic infrastructures will need careful consideration and clarification for the future of women's rights.
